# CXCL14 facilitates the growth and metastasis of ovarian carcinoma cells via activation of the Wnt/β-catenin signaling pathway

**DOI:** 10.1186/s13048-021-00913-x

**Published:** 2021-11-17

**Authors:** Li-Na Gao, Man Hao, Xiao-Hui Liu, Li Zhang, Yan Dong, Yu-Fang Zhang, Xiao-Chun He

**Affiliations:** The Second Obstetrics Department, Gansu Provincial Maternity and Child-care Hospital, No. 143 North Qilihe Street, Qilihe District, Lanzhou, Gansu China

**Keywords:** CXCL14, Ovarian cancer, EMT, Wnt/β-catenin

## Abstract

**Background:**

There is an urgent need to identify potential targets in anticancer therapy to improve the survival and prognosis of patients with ovarian cancer (OC). Herein, we investigated the functional significance of chemokine (C-X-C motif) ligand 14 (CXCL14) in OC cell growth and epithelial–mesenchymal transition (EMT).

**Methods:**

qRT PCR and western blotting was used to detect CXCL14 mRNA level and protein expression, respectively. The functional mechanism of CXCL14 in OC was investigated by CCK-8, colony formation and transwell assays. The migration ability of OC cell was determined using wound healing. The protein expressions of CXCL14 and β-catenin in OC tissues were determined by immumohistochemical staining.

**Results:**

We demonstrated that high levels of CXCL14 were associated with a worse prognosis in patients with OC. CXCL14 knockdown considerably restrained the growth, migration and invasion of OC cell in vitro. In contrast, ectopic CXCL14 overexpression yielded the opposite results. Investigations to determine the underlying molecular mechanisms revealed that the Wnt/β-catenin signaling pathway is involved in CXCL14-facilitated OC cell invasiveness.

**Conclusion:**

These data collectively demonstrate that CXCL14 contributes to OC cell growth and metastatic potential by regulating the Wnt/β-catenin signaling pathway.

## Introduction

Ovarian cancer (OC) is the most lethal cancer among gynecologic malignancies [1]. Despite considerable improvements in surgery and chemoradiotherapy, survival rate remains deplorable owing to recurrences and metastases [2]. Recent clinical and experimental evidence demonstrates that epithelial–mesenchymal transition (EMT) is a critical event in the process of cancer cell metastasis [3]. EMT is a transdifferentiation process, in which epithelial cells undergo changes and demonstrate mesenchymal phenotypes, and is mainly characterized by the gain of the mesenchymal biomarker, N-cadherin, and the loss of the epithelial biomarker, E-cadherin [4].

CXCL14 is a key homeostatic chemokine that facilitates immune surveillance of skin epithelium through recruitment of various immune cell types, including natural killer cells, dendritic cells (DCs), and T cells [5]. A study has investigated the abnormal expression of CXCL14 and its biological functions in diverse carcinomas [6]. Furthermore, CXCL14 has been demonstrated to have both tumor-suppressive and pro-oncogenic roles. For example, CXCL14 exerts anticancer effects in head and neck cancer, colorectal carcinoma, and oral cancer [7, 8]. Moreover, CXCL14 expression is downregulated in breast cancer, and CXCL14 overexpression inhibits cell growth and invasion in vitro and attenuates tumor growth and pulmonary metastasis of breast carcinoma cells in vivo [9]. However, CXCL14 expression is higher in glioblastoma tissues than in surrounding healthy tissues, and CXCL14 enhances the migration and proliferation of glioblastoma cells [10].

Earlier investigations have closely linked chemokines and their membrane receptors to the pathological EMT process [11]. For instance, Wang demonstrated that C-C motif chemokine-17 (CCL-17) was secreted from CXCL14-activated fibroblasts as a regulatory factor of the CXCL14-triggered breast carcinoma cell EMT and metastasis [12]. Additionally, CXCL14 was identified to function as a chemokine for the M2-type macrophage, which triggers EMT [13]. Upregulated CXCL14 expression is associated with poor overall survival of patients, as it promotes OC cell proliferation [14]. However, the role of CXCL14 in EMT and the metastasis of OC, as well as the molecular mechanism, has not been elucidated.

In this study, we evaluated the expression of CXCL14 in patients with OC and determined its correlation with the clinical outcomes of patients. Furthermore, we explored the functional significance of CXCL14 expression in OC cell invasion and EMT.

## Materials and methods

### Cell culture

The human ovarian surface epithelial cell line HOSEpiC and four ovarian carcinoma cell lines (OVCAR3, SKOV3, A2780, and CAOV3) were purchased from Nanjing KeyGen Biotech (Nanjing, China). Cells were cultured in DMEM (Thermo Fisher Scientific, Waltham, MA, USA) supplemented with 10% FBS, 100 U/ml penicillin and 0.1 mg/ml streptomycin (Gibco, Carlsbad, CA, USA) at 37 °C in an incubator with 5% CO_2_. The inhibitor of Wnt/β-catenin signaling, XAV939, was purchased from Selleck Chemicals (Shanghai, China).

### Tissue samples

Fifty patients with ovarian cancers and 50 patients with benign epithelial ovarian tumor were obtained at operation in the Gansu Provincial Maternity and Child-care Hospital. OC tissues (*n* = 50) and non-tumor ovarian tissues (n = 50, normal ovarian tissues more than 5 cm away from the benign epithelial ovarian tumor’s margin) were acquired from OC patients or patients with benign epithelial ovarian tumor, respectively [15]. Written informed consent was obtained from all the patients. Paraffin-embedded tissues were cut into 4-μm-thick sections. The immunohistochemistry procedure to determine CXCL14 and β-catenin expression was performed as described previously [16, 17]. To evaluate CXCL14 or β-catenin staining, its extent was scored by assigning a staining percentage to positive tumor cells (0, none; 1,< 20% of positive staining cells; 2, 20–50% of positive staining cells; 3,> 50% of positive staining cells). Samples with scores of 0, 1, or 2 were defined as low expression, while cases with a score of 3 were defined as having high expression. The study was approved by the Medical Ethical Committee of Gansu Provincial Maternity and Child-care Hospital.

### Plasmid transfections

CXCL14 shRNA (sh-CXCL14 #1, sh-CXCL14 #2) and shRNA negative control (sh-NC) were obtained from GeneCopoeia (Guangzhou, China). pcDNA3.1-CXCL14 (CXCL14-OE) and pcDNA3.1-empty vector (Vector) were purchased from GenePharma (Shanghai, China). Transfection or co-transfection was performed using the Lipofectamine 3000 kit (Thermo Fisher Scientific) according to the manufacturer’s instructions.

### RNA extraction and quantitative real-time PCR (qRT-PCR)

RNA was extracted from cells using the TRIzol® kit (Thermo Fisher Scientific). First-strand cDNA was obtained using the PrimeScript® RT Kit (Takara, Dalian, China). Then, qRT-PCR was conducted using a SYBR Green Real-time kit (TaKaRa) on an IQ5 real-time PCR system (Bio-Rad). The following primers were used: CXCL14 forward, 5′-CGCTACAGCGACGTGAAGAA-3′ and reverse, 5′-GTTCCAGGCGTTGTACCAC-3′; β-catenin forward, 5′-ATGGAGCCGGACAGAAAAGC-3′ and reverse, 5′-CTTGCCACTCAGGGAAGGA-3′; MMP-7 forward, 5′-GAGTGAGCTACAGTGGGAACA-3′ and reverse, 5′-CTATGACGCGGGAGTTTAACAT-3′; Cyclin D1 forward, 5′-GCTGCGAAGTGGAAACCATC-3′ and reverse, 5′-CCTCCTTCTGCACACATTTGAA-3′; Axin2 forward, 5′-TGACTCTCCTTCCAGATCCCA-3′ and reverse, 5′-TGCCCACACTAGGCTGACA-3′; and GAPDH forward, 5′-TGGATTTGGACGCATTGGTC-3′ and reverse, 5′-TTTGCACTGGTACGTGTTGAT-3′. Quantification was performed using the 2^−ΔΔCt^ method. GAPDH was used as the internal control.

### Cell proliferation assay

Cell proliferation was determined using a Cell Counting Kit-8 (CCK-8) kit (Dojindo, Tokyo, Japan). Cells were collected on days 1, 2, 3, and 4. After incubation with CCK-8 reagent for 2 h, the absorbance (OD) was measured using a microplate reader (Bio-Tek, USA) at 450 nm.

### Colony formation assay

One thousand cells were maintained in 6-well plates for 2 weeks in an incubator at 37 °C. Then, the cell colonies were fixed using 4% polyoxymethylene and stained with 1% crystal violet (Beyotime, Nanjing, China). The number of clones with more than 50 cells was counted.

### Migration assay

Cells (1 × 10^4^) were seeded into 6-well plates overnight to form a complete monolayer. Then, a 200 μl pipette tip was used to make wounds across the cell monolayer. After removing dead cells with PBS, cells were cultured with serum-free DMEM medium for 24 h. Each wound was photographed at 0 h or 24 h, and the wound healing rate was calculated using the following formula: migration rate = (0 h scratch width − 24 h scratch width)/0 h scratch width) × 100%.

### Invasion assay

The upper basement membrane of an 8-μm pore transwell chamber (Corning, NY, USA) was pre-coated with 20 μg Matrigel. The cell suspension (200 μL, 1 × 10^3^) was plated in the upper compartment, and the lower compartment was filled with DMEM (600 μL) supplemented with 20% FBS. After 48 h, the invading cells were fixed with formaldehyde and stained with 1% crystal violet. The number of invading cells in five random visual fields was counted using a microscope.

### Immunoblotting

Total protein was extracted and separated using 10% SDS-PAGE. To obtain nuclear and cytoplasmic fractions, subcellular fractionation was performed as described previously [18]. After transfer onto PVDF membranes (Millipore, Braunschweig, Germany), the membranes were incubated with antibodies against β-catenin, E-cadherin, N-cadherin, cyclin D1, MMP-7, Axin2, LaminB, or GAPDH (1:1000, Abcam, Cambridge, UK). Then, the membranes were incubated with a secondary antibody (Beyotime Institute of Biotechnology) for 2 h. The bands were detected using the BeyoECL Plus kit (Beyotime).

### Statistical analyses

The data were analyzed using GraphPad Prism 8 software. They are shown as the mean ± SD. Significant differences were evaluated using the Student’s *t*-test or one-way analysis of variance (ANOVA) with the Bonferroni post-hoc test. Statistical significance was set at *P* < 0.05.

## Results

### The elevation of CXCL14 and its prognostic implication in OC

**Fig. 1:**
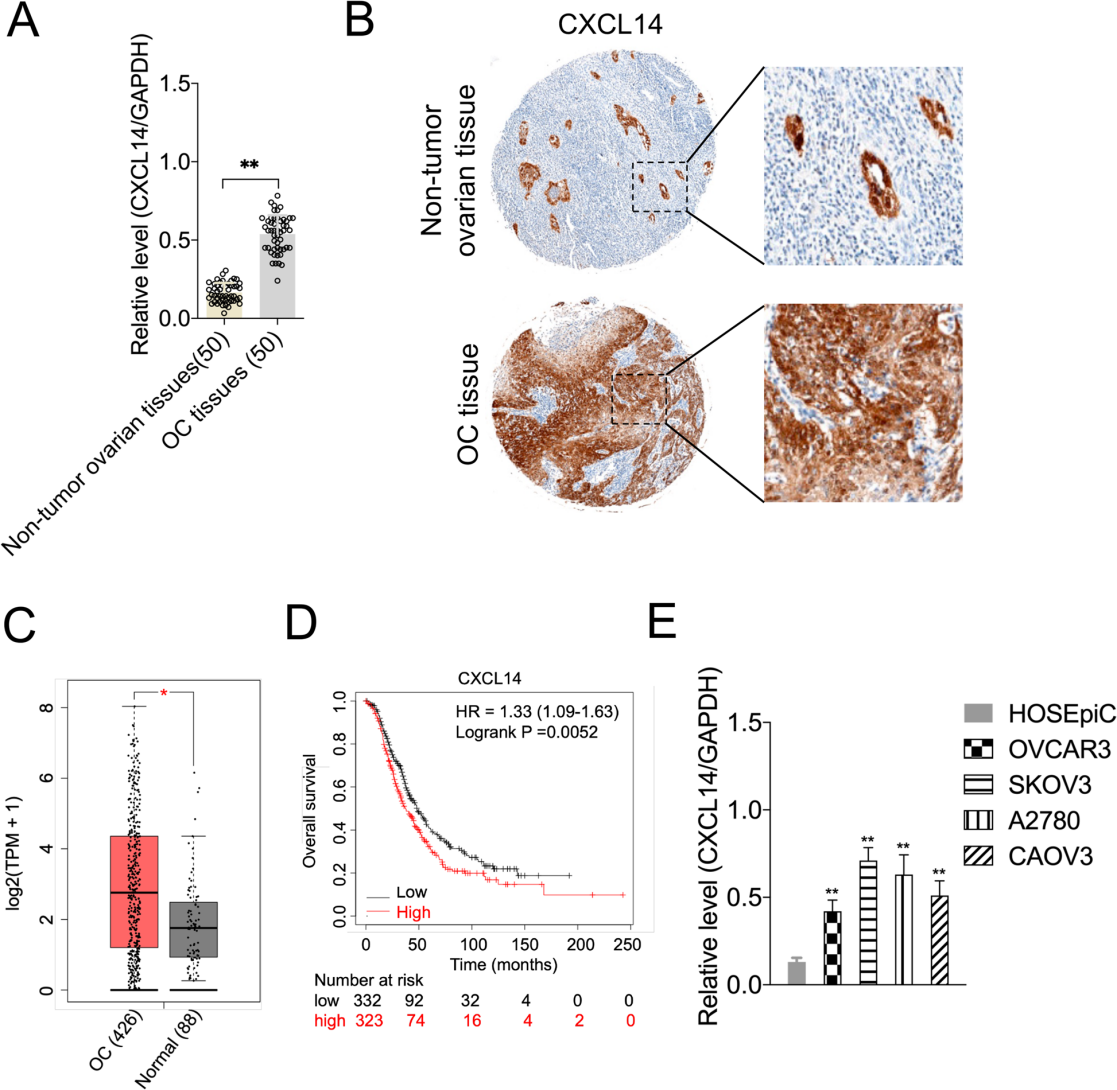
Relative CXCL14 expression in OC tissues and cell lines. **A** The qRT-PCR analysis of CXCL14 mRNA expression in 50 paired specimens of OC tissues versus non-tumor ovarian tissues. ^**^P < 0.01 compared with non-tumor ovarian tissues. **B** Representative images of CXCL14 staining in surgical specimens from OC tissues and non-tumor ovarian tissues (original magnification, × 100). **C** The expression of CXCL14 in ovarian cancer (*n* = 426) and normal (*n* = 88) determined by GEPIA database. ^*^P < 0.05, Y axis indicates relative expression value, log2(TPM + 1). TPM = Transcript per million. **D** Correlation between OC patient survival and CXCL14 expression (probe: 222484_s_at) was analyzed by Kaplan-Meier analysis (http://kmplot.com/analysis/index.php?p=service&cancer=ovar). Cutoff value =203. **E** The qRT-PCR analysis of CXCL14 mRNA expression in OC cell lines and normal cell line, HOSEpiC. ^**^P < 0.01 compared with HOSEpiC

To investigate the dysregulation of CXCL14 expression in OC cells, we measured the mRNA and protein levels of CXCL14 in 50 pairs of OC tissues and non-tumor ovarian tissues. As presented in Fig. 1A, the mRNA levels of CXCL14 were higher in OC specimens than in non-tumor ovarian tissues. Similarly, CXCL14 expression was significantly higher in OC (84%, 42 in 50 cases) relative to non-tumor ovarian tissues, which was demonstrated using immunohistochemical staining (Fig. 1B). Moreover, we did not find any difference in CXCL14 expression between the serous (*n* = 34) and mucinous (*n* = 12) subtypes. The Cancer Genome Atlas (TCGA) and the Genotype-Tissue Expression (GTEx) projects were used to investigate the expression of CXCL14 in OC cells using GEPIA (http://gepia.cancer-pku.cn/) [19]. As illustrated in Fig. 1C, CXCL14 was substantially upregulated in OC cells (*n* = 426) as compared to that in normal tissues (*n* = 88). Next, an analysis of the clinical characteristics of patients with OC revealed that CXCL14 was associated with histological grade, pT/pN/pM status, and FIGO stage (*P* < 0.05, Table 1). Remarkably, Kaplan–Meier survival analyses (http://kmplot.com/analysis/index.php?p=service&cancer=ovar) indicated that OC patients with higher CXCL14 expression exhibited a shorter OS (patients were divided into high- and low-group based on the mean value. Cutoff = 203; Fig. 1D). Finally, CXCL14 expression in OC cells (A2780, OVCAR3, CAOV3, and SKOV3) was relatively higher than that in the normal cell line, HOSEpiC (Fig. 1E). These findings indicate that CXCL14 is upregulated in OC, with higher levels correlating to worse OS.

**Table 1:** Association of CXCL14 expression with patient’s clinico-pathological features in ovarian carcinomas

Clinical parameter	CXCL14			***P***-value^*^
	All cases	Low expression	High expression	
**Age (years)**				0.414
≤ 51	16	7	9	
> 51	34	20	14	
**Histological type**				0.348
Serous	34	20	14	
Mucinous	12	5	7	
Others^a^	4	2	2	
**pT status**				0.024
pT1	20	13	7	
pT2	13	8	5	
pT3	17	6	11	
**pN status**				0.001
pN0	28	19	9	
pN1	22	8	14	
**pM status**				0.018
pMX	34	22	12	
pM1	16	5	11	
**FIGO stage**				0.029
I	9	7	2	
II	13	8	5	
III	18	8	10	
IV	10	4	6	

*Chi-square test

^a^Endometrioid, clear cell and undifferentiated types

*FIGO* International Federation of Gynecology and Obstetrics

### Silencing of CXCL14 inhibits OC cell growth

**Fig. 2:**
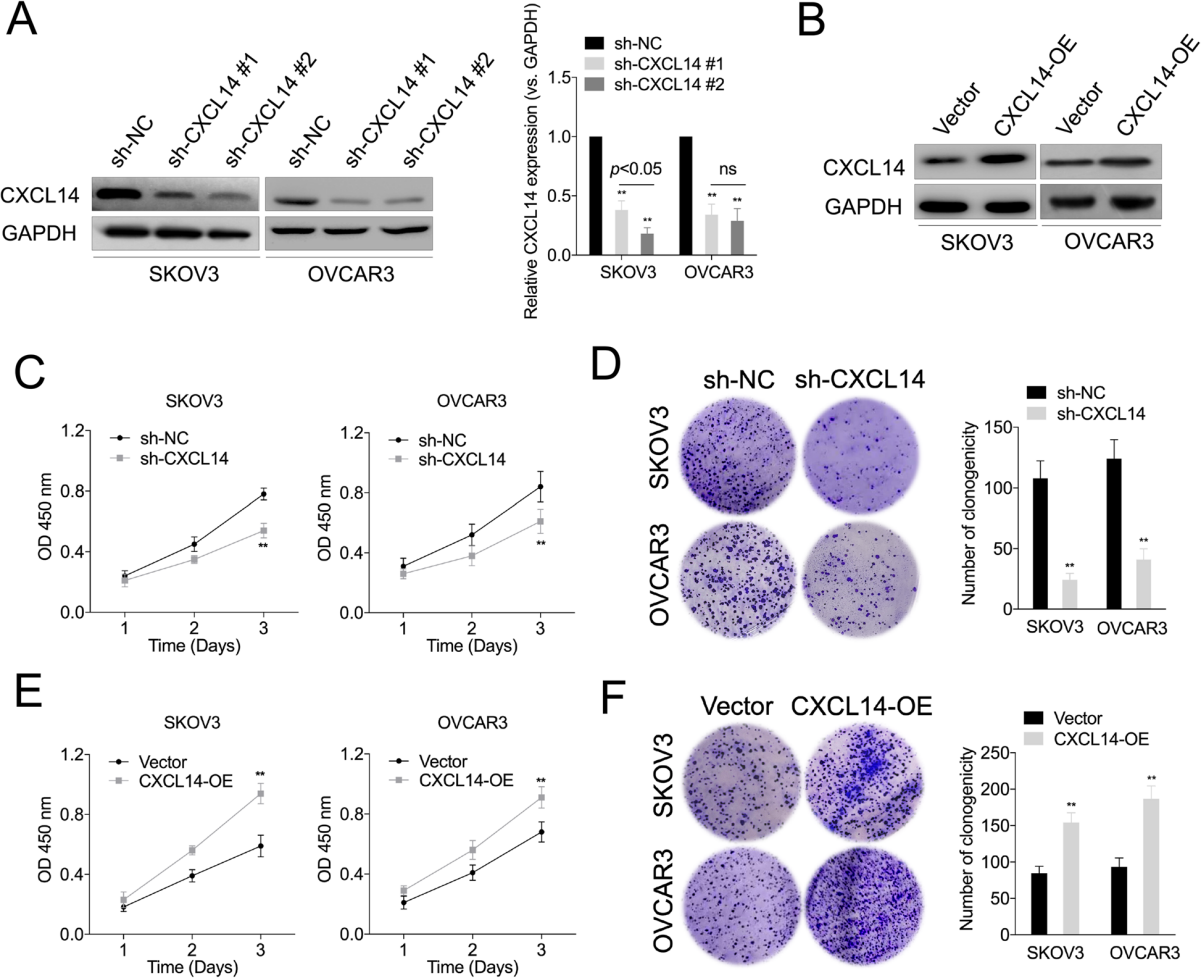
The effects of CXCL14 knockdown or overexpression on OC cell proliferation. **A** Western blot analyses of CXCL14 expression in SKOV3 and OVCAR3 cells transfected with the CXCL14-shRNA (sh-CXCL14 #1, sh-CXCL14 #2) or shRNA negative control (sh-NC). Histogram represents the quantitative analysis of gray ratio. **B** Western blot analyses of CXCL14 expression in SKOV3 and OVCAR3 cell transfected with the CXCL14 overexpressing plasmid (CXCL14-OE) or vector. **C** The effect of CXCL14 silencing on SKOV3 and OVCAR3 cell growth. **D** The effect of CXCL14 silencing on OC cell colony formation. Y-axis represents the number of the number of colonies. (E) The effect of CXCL14-overexpression on SKOV3 and OVCAR3 cell growth using the CCK-8 assay. (F) The effect of CXCL14-overexpression on SKOV3 and OVCAR3 cell growth using the colony formation assay. ^**^P < 0.01 compared with sh-NC or vector

To illustrate the biological function of CXCL14 in OC, gain- and loss-of-function assays were performed through the introduction of CXCL14 into OC cell and knockdown of CXCL14 in OC cell (Fig. 2A and B). Although there was a slightly different silencing efficiency between sh-CXCL14 #1 and sh-CXCL14 #2 in OVCAR3 cell, the difference was not significant. However, sh-CXCL14 #2 produced a more efficient silencing effect in SKOV3 cell. After comprehensive consideration, sh-CXCL14 #2 was selected for functional studies. CXCL14 knockdown noticeably suppressed OC cell growth, as evidenced by CCK-8 (Fig. 2C) and colony formation assays (Fig. 2D). In contrast, ectopic overexpression of CXCL14 in OC cell yielded opposite results (Fig. 2E and F). Collectively, these findings suggest that CXCL14 serves as an oncogene and facilitates OC cell growth.

### Silencing of CXCL14 inhibits OC cell migration, invasion and EMT

**Fig. 3:**
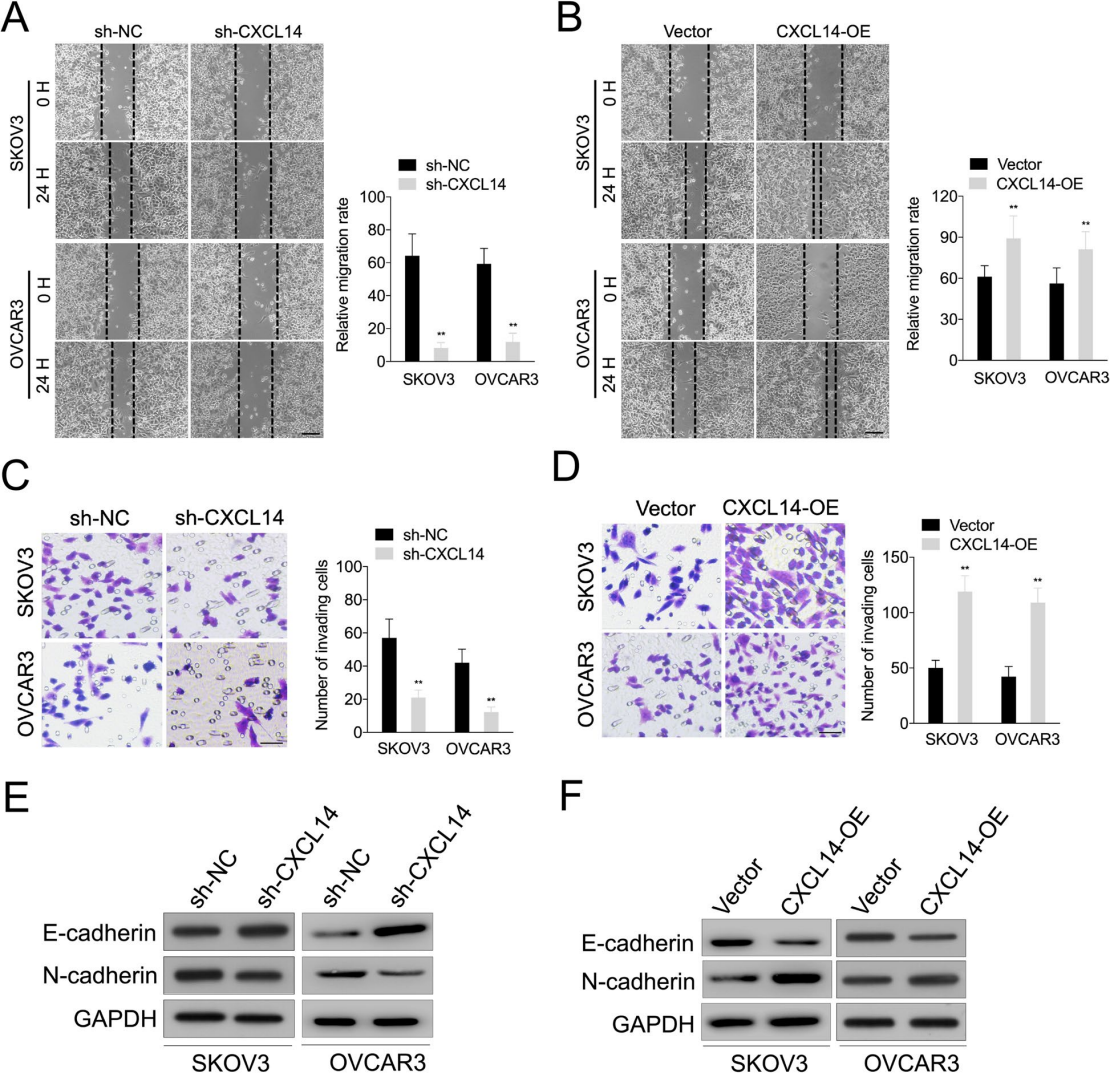
The effects of CXCL14 on OC cell invasion and EMT. **A**-**B** The effects of CXCL14 downregulation or upregulation on the migration capabilities of OC cell using the wound healing assay. Scale bar: 200 μm. **C**-**D** The effects of CXCL14 downregulation or upregulation on the invasive capabilities of OC cells using the Transwell assay. Scale bar: 200 μm. ^**^P < 0.01 compared with sh-NC or vector. **E** Western blot analyses of the expression levels of E-cadherin and N-cadherin in sh-CXCL14 transfected OC cell. **F** Western blot analyses of the expression levels of E-cadherin and N-cadherin in CXCL14 overexpressing OC cell

Next, we investigated the effect of CXCL14 on OC cell invasiveness and the EMT process. Firstly, the wound healing assay exhibited that CXL14 deletion restrained the migratory capacity of OC cell; however, upregulation of CXL14 promoted cell migration (Fig. 3A and B). Transwell assay also revealed that CXCL14 knockdown suppressed the invasive ability of OC cell, whereas CXCL14 upregulation increased OC cell invasion (Fig. 3C and D). The expression of E-cadherin was increased in two cell lines after silencing of CXCL14, whereas N-cadherin expression was decreased (Fig. 3E). Nevertheless, the epithelial marker, E-cadherin, was downregulated in the OC cell after CXCL14 upregulation, and the protein expression of the mesenchymal marker N-cadherin was increased (Fig. 3F). These data suggest that CXCL14 promotes the EMT and invasion of OC cell.

### Wnt/β-catenin signaling is involved in CXCL14-mediated OC cell growth and invasiveness

**Fig. 4:**
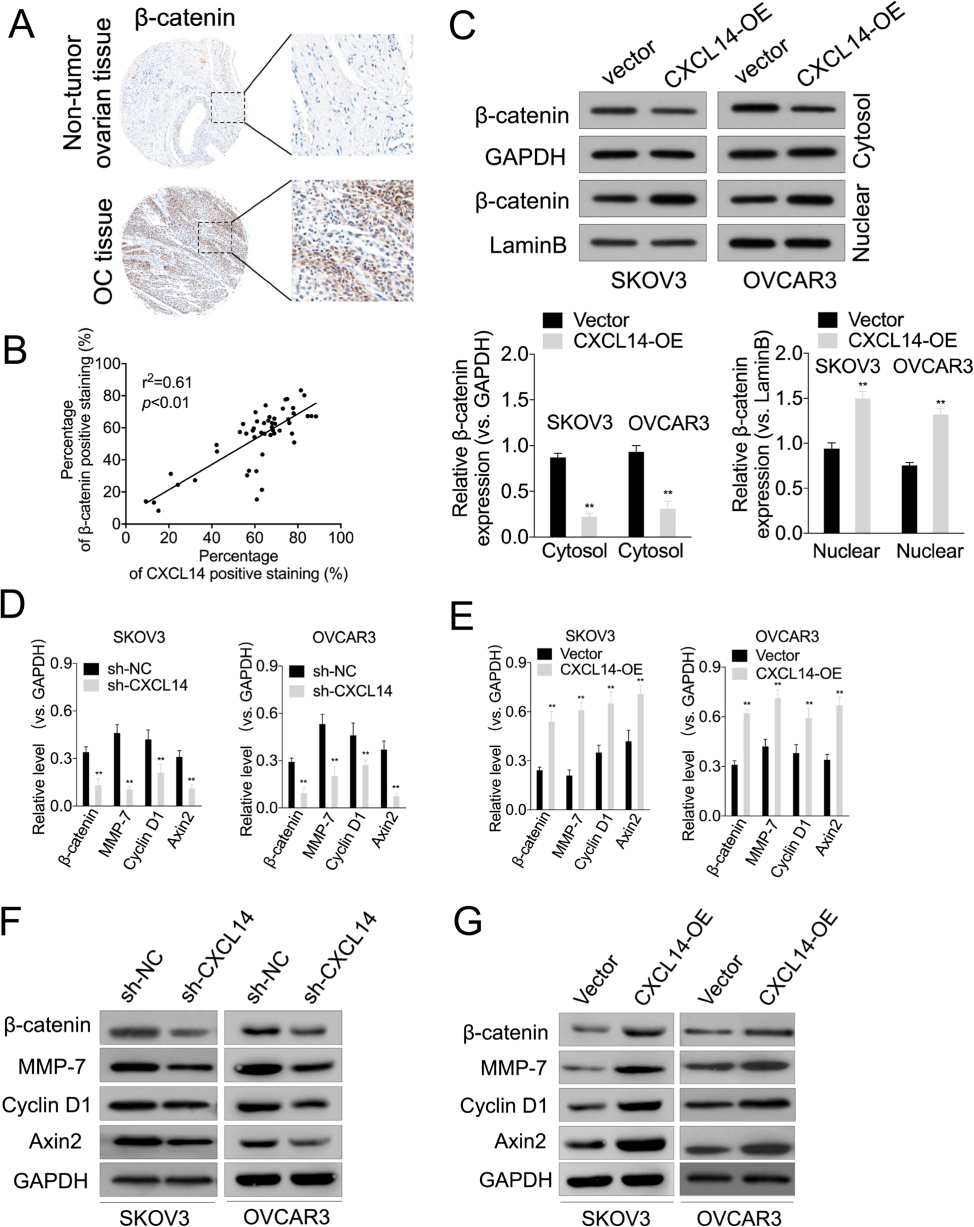
The effects of CXCL14 knockdown or overexpression on Wnt/β-catenin signaling-related markers. **A** Representative images of immunohistochemical staining of β-catenin in surgical specimens from OC tissues and non-tumor ovarian tissues (original magnification, × 100). **B** Correlation study of CXCL14 and β-catenin in OC tissues. Pearson correlation coefficient were used for statistical analysis of the correlation between CXCL14 and β-catenin. **C** Western blotting shows that overexpressing CXCL14 in OC cells increased the nuclear accumulation of β-catenin, respectively. ^**^P < 0.01 compared with vector. **D**-**E** Following CXCL14 shRNA or overexpression treatment, qRT-PCR detection of the mRNA levels (cyclin D1, MMP-7, Axin2) in OC cells. ^**^P < 0.01. **F**-**G** Following CXCL14 shRNA or overexpression treatment, western blotting detection of the protein expressions of cyclin D1, MMP-7 and Axin2 in OC cells. ^**^P < 0.01 compared with sh-NC or vector

The Wnt/β-catenin signaling axis is tightly regulated by β-catenin, which can trigger the transcriptional activation of several metastasis-related genes. To determine the clinical relevance of CXCL14-β-catenin signaling in OC, we performed immunohistochemical staining for β-catenin in OC tissues. As shown in Fig. 4A, β-catenin expression was significantly higher in OC (72%, 36 in 50 cases) relative to non-tumor ovarian tissues. Moreover, a positive correlation between β-catenin and CXCL14 protein levels (Fig. 4B, *P* < 0.01, r^2^ = 0.61) was observed in these OC tissues. Nuclear accumulation of β-catenin is the central event in the activation of the Wnt/β-catenin signaling pathway. Next, we investigated whether CXCL14 can regulate β-catenin nuclear accumulation in OC cells. As shown in Fig. 4C, overexpression of CXCL14 enhanced the nuclear β-catenin level in SKOV3 and OVCAR3 cell. Next, we detected the changes in Wnt/β-catenin downstream genes in CXCL14 knockdown or overexpressing OC cell. CXCL14 knockdown reduced the mRNA and protein levels of β-catenin, MMP-7, cyclin D1, and Axin2 in OC cells. In contrast, CXCL14 overexpression upregulated the mRNA and protein levels of β-catenin, Axin2, MMP-7, and cyclin D1 in OC cell (Fig. 4D–G).

**Fig. 5:**
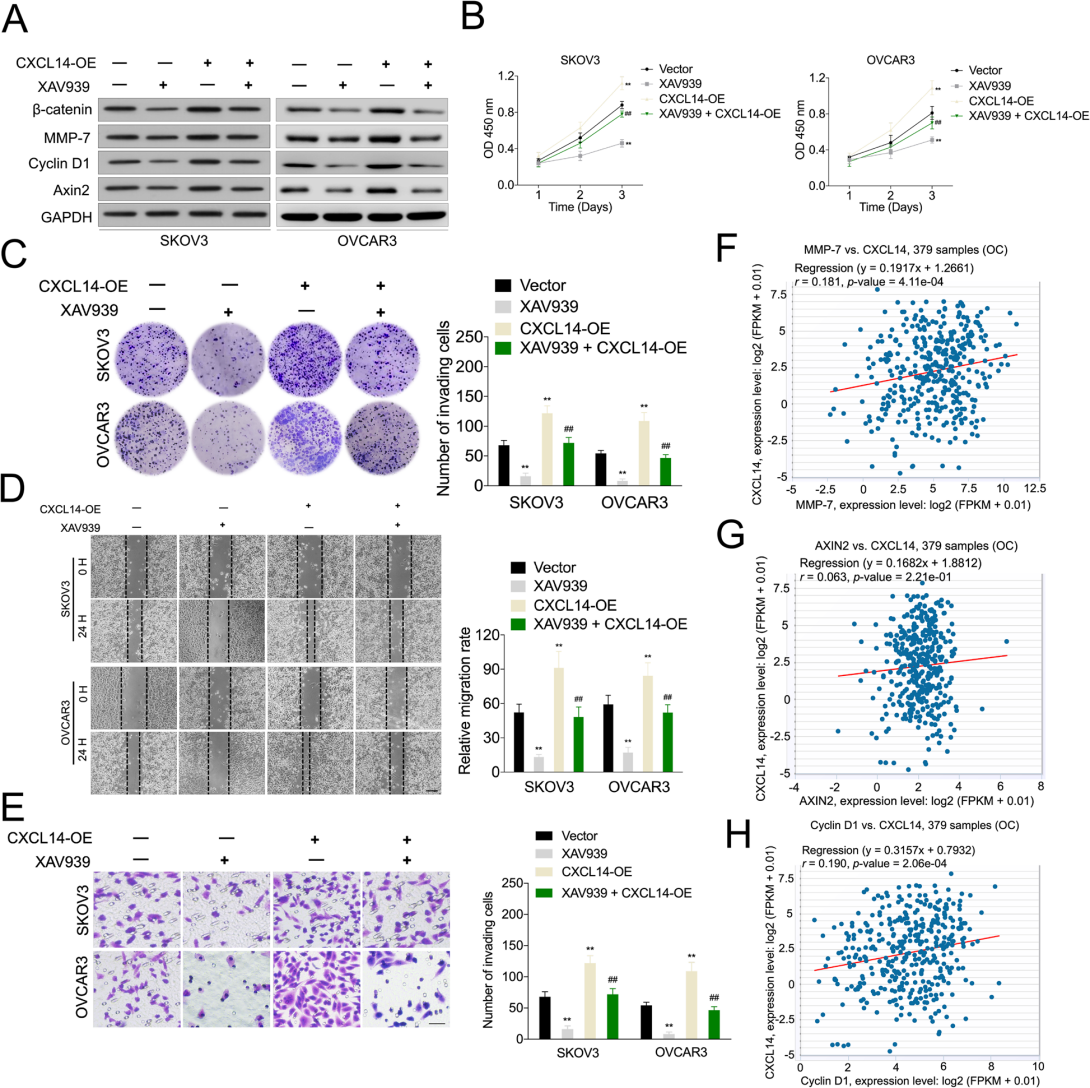
Wnt/β-catenin signaling is involved in CXCL14-facilitated ovarian OC cell EMT and invasion. **A** Western blot analysis of the indicated expression levels (β-catenin, cyclin D1, MMP-7, Axin2) in CXCL14 overexpressing SKOV3 and OVCAR3 cells treated with XAV939 (10 μM). **B** Determination of the proliferative capability of CXCL14 overexpressing SKOV3 and OVCAR3 cells treated with XAV939 using the CCK-8. **C** Determination of the proliferative capability of CXCL14 overexpressing SKOV3 and OVCAR3 cells treated with XAV939 using the colony formation assay. **D** Determination of the migration capability of OC cells using the wound healing assay. Scale bar: 200 μm. **E** Determination of the invasive capability of OC cells using the Transwell assay. Scale bar: 200 μm. ^**^P < 0.01 compared with vector, ^##^*P* < 0.01 compared with CXCL14-OE. **F**-**H** Statistical analysis of the correlation between CXCL14 and Wnt/β-catenin target genes (Cyclin D1, MMP-7, Axin2) in OC patients (*n* = 379) cohort using TCGA database

To determine whether Wnt/β-catenin signaling was involved in CXCL14-induced OC cell metastasis, the specific inhibitor of Wnt/β-catenin signaling, XAV939, was utilized. XAV939 (10 μM) treatment decreased the expression levels of MMP-7, cyclin D1, and Axin2 in CXCL14 overexpressing OC cells (Fig. 5A) [20]. Additionally, XAV939 suppressed CXCL14-facilitated OC cell growth in the CCK-8 (Fig. 5B) and clonogenic assays (Fig. 5C). Similarly, XAV939 suppressed CXCL14-facilitated OC cell migration and invasion in the wound healing and transwell assay, respectively (Fig. 5D-E). Finally, we analyzed the correlation between CXCL14 and Wnt/β-catenin target genes in OC using TCGA dataset. The result of Pearson’s correlation analysis revealed that CXCL14 expression was positively correlated with Axin2, cyclin D1, and MMP-7 levels in OC tissues (Fig. 5F–H). These observations indicated that Wnt/β-catenin signaling was responsible for CXCL14-induced OC cell growth and invasion.

## Discussion

Emerging evidence has established that CXCL14 is important for cell growth, invasion, metastasis, and chemoresistance in multiple cancers [21]. Although it has been reported that upregulated CXCL14 expression promotes OC proliferation, little is known about its biological role in the various pathological processes of OC, including EMT and invasion [14]. Herein, we demonstrated that the CXCL14 level was elevated in carcinoma tissues as compared with para-cancerous tissues. Importantly, high CXCL14 expression is associated with metastasis and poor prognosis. Next, our results indicated that CXCL14 knockdown suppressed OC cell growth, colony formation, and metastatic phenotypes, whereas CXCL14 overexpression produced the opposite results. Further analyses revealed that the canonical Wnt/β-catenin signaling is required for CXCL14-mediated OC cell growth, migration and invasion.

Cancer metastasis is a complicated process, and cancer cells need to acquire metastatic characteristics, such as decreased adhesiveness and increased migration and invasive ability, to escape the confines of the primary tumor site and establish distant metastases [22]. In addition to generating cancer stem cells and resulting in treatment resistance, EMT is considered to be necessary in the initial events of the metastasis cascade by conferring an aggressive phenotype [23]. Thus, the reversal of cancer cell EMT represents a considerable potential therapeutic option for patients with OC.

Importantly, Wnt/β-catenin signaling was involved in the CXCL14-induced EMT process. First, the present findings revealed that reduced CXCL14 expression obstructed EMT, whereas upregulation of CXCL14 contributed to EMT, thus indicating that CXCL14 acts as a crucial regulatory factor of EMT in OC cells. Moreover, β-catenin is a vital element in the Wnt signaling pathway, and β-catenin facilitates the transcriptional activation of EMT-associated genes, including cyclin D1, MMP-7, and Axin2. Additionally, CXCL14 knockdown in SKOV3 cells lowered the expression of Wnt/β-catenin-associated genes, whereas CXCL14 overexpression in OVCAR3 cells augmented their expression. To verify whether CXCL14 triggers OC cell growth and EMT via the Wnt/β-catenin signaling pathway, OC cells were treated with XAV939. Intriguingly, inhibition of Wnt/β-catenin signaling attenuated CXCL14-induced OC cell EMT, as well as cell growth and invasion. Thus, our data suggest that Wnt/β-catenin signaling is necessary for CXCL14-facilitated OC cell growth and invasion.

In tumor tissue cells, owing to various factors, the activation of signal transducer and activator of transcription 3 (STAT3) appears to be in a persistently and abnormally high expression state, which leads to abnormal proliferation of malignant tumor cells. Previous studies have shown that overexpression of CXCL14 increases the phosphorylation level of STAT3 in OC cells, thereby promoting the activation of the STAT3 signaling pathway [14]. Moreover, it is indicated that STAT3 phosphorylation is involved in EMT and tumor metastasis. Specifically, several molecules have been demonstrated to promote tumor metastasis by activating the STAT3 signaling pathway [24, 25]. Accordingly, suppression of STAT3 phosphorylation downregulated the expression of mesenchymal markers (N-cadherin, Snail, and MMP-9) and increased the levels of the epithelial marker, E-cadherin [26]. However, additional investigations are required to identify whether STAT3 is involved in the CXCL14-facilitated OC cell invasion and EMT processes.

It is well recognized that nuclear β-catenin is associated with transcription factor 4 (TCF4) to activate target gene transcription [27]. The key molecular events in Wnt signaling are nuclear β-catenin accumulation and β-catenin/TCF4 complex transcriptional activity [28]. Nuclear β-catenin binds to members of the TCF/LEF family of transcription factors, including Twist and Snail1/2, to trigger EMT [29, 30]. Although β-catenin serves as a downstream effector in CXCL14-induced OC cell growth and invasion, it deserves further exploration of the complex interaction of β-catenin and CXCL14. To gain more insights into CXCL14-induced β-catenin transcriptional activity, the physical interaction between CXCL14 and β-catenin may be worth exploring in future studies. Furthermore, chemokines exert their effects by binding to specific transmembrane receptors. To date, several CXCL14 receptors have been identified, including insulin-like growth factor 1 receptor (IGF-1R) and C-X-C Motif Chemokine Receptor 4 (CXCR4) [13, 31]. Chemokine receptors have been reported to trigger the process of EMT and play an important role in cancer development [32, 33]. Further works are also needed to investigate which chemokine receptor plays the principal role in CXCL14-inducing OC cell EMT.

## Conclusion

**Fig. 6:**
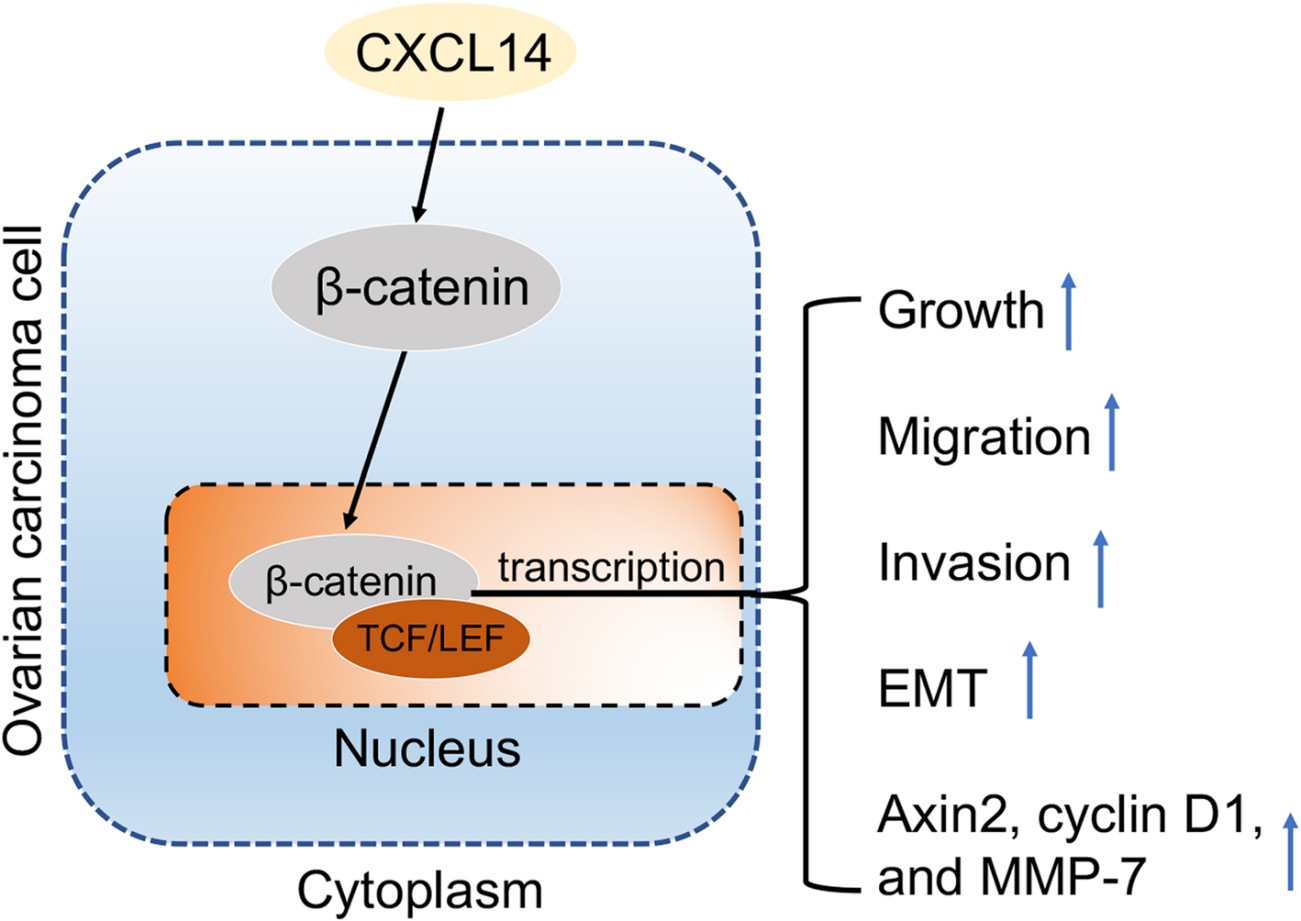
Schematic diagram of the regulatory mechanism of the CXCL14/Wnt/β-catenin signaling axis in promoting ovarian cancer cells migration, invasion and EMT

Altogether, our study demonstrates that high CXCL14 expression is associated with increased lymph node metastasis and worse prognosis in patients with OC. In conclusion, we found that CXCL14 promotes OC cell EMT and the metastatic phenotype by influencing the Wnt/β-catenin signaling pathway (Fig. 6).

## Data Availability

The datasets generated during the current study are available from the corresponding author on reasonable request.

## Abbreviations

CXCL14: Chemokine (C-X-C motif) ligand 14
EMT: Epithelial–mesenchymal transition
OC: Ovarian cancer
qRT-PCR: Quantitative real-time PCR
CCK-8: Cell Counting Kit-8
TCGA: The Cancer Genome Atlas
STAT3: Signal transducer and activator of transcription 3
